# Comparison between Conditioned Pain Modulation Paradigms Using Cold Pressor Conditioning Stimulus versus Ischemic Pressure Stimulus in Women with Fibromyalgia and Its Impact on Clinical Status: A Cross-Sectional Study

**DOI:** 10.3390/biomedicines12102222

**Published:** 2024-09-29

**Authors:** Víctor Riquelme-Aguado, María Elena González-Álvarez, Alazne Zabarte-Del Campo, Josué Fernández-Carnero, Antonio Gil-Crujera, Francisco Gómez-Esquer, Jorge Hugo Villafañe

**Affiliations:** 1 Department of Basic Health Sciences, Rey Juan Carlos University, 28933 Madrid, Spain; antonio.gil@urjc.es (A.G.-C.); francisco.gomez.esquer@urjc.es (F.G.-E.); 2 Grupo de Investigación Consolidado de Bases Anatómicas, Moleculares y del Desarrollo Humano de la Universidad Rey Juan Carlos (GAMDES), 28922 Alcorcón, Spain; 3 Fisioterapia Oreka CB, 45200 Illescas, Spain; alazne_1990@hotmail.es; 4 Cognitive Neuroscience, Pain and Rehabilitation Research Group (NECODOR), Faculty of Health Sciences, Rey Juan Carlos University, Alcorcón, 28922 Madrid, Spain; josue.fernandez@urjc.es; 5 Escuela Internacional de Doctorado, Rey Juan Carlos University, 28008 Madrid, Spain; 6 Department of Physical Therapy, Occupational Therapy, Rehabilitation and Physical Medicine, Rey Juan Carlos University, 28032 Madrid, Spain; 7 Musculoskeletal Pain and Motor Control Research Group, Faculty of Sport Sciences, Universidad Europea de Madrid, 28670 Villaviciosa de Odón, Spain; mail@villafane.it; 8 Department of Physiotherapy, Faculty of Sport Sciences, Universidad Europea de Madrid, 28670 Villaviciosa de Odón, Spain

**Keywords:** fibromyalgia, conditioned pain modulation, central pain, central sensitization, cold pressor test, ischemic pressure

## Abstract

**Background/Objectives:** Fibromyalgia (FM) is a syndrome characterized by widespread chronic pain as the primary symptom. Neurophysiological pain mechanisms, such as the function of the descending inhibitory system, are impaired in this condition. The main objective of this study was to compare the results of two paradigms to evaluate CPM in women with FM. The secondary objective was to correlate the results of each CPM paradigm with the clinical status of patients with FM. **Methods:** One hundred and three FM women were divided into two groups: fifty patients diagnosed with FM were assigned to the conditioned pain modulation (CPM) group using a cold pressor stimulus, and fifty-three patients were assigned to the CPM group using the ischemic pressure stimulus. The main outcome measures were pain intensity, disability, mechanical hyperalgesia, and CPM. **Results:** The primary analysis revealed significant differences between the results obtained from the different CPM protocols. Poorer outcomes in the cold pressor test correlated with higher pain intensity and a greater disability index. **Conclusions:** Pain modulation abnormalities in FM patients were evident when using either the cold pressor or ischemic pressure stimuli to establish the CPM paradigm. The cold pressor conditioning stimulus elicited a stronger response than the ischemic pressure stimulus in FM patients.

## 1. Introduction

Fibromyalgia (FM) is a condition characterized by widespread chronic pain as its primary symptom, accompanied by other significant symptoms such as chronic fatigue, stiffness, psychological issues, and sleep disturbances [1,2,3,4]. In Madrid, Spain, the prevalence of FM in women is estimated to affect up to 5% of the population [5]. According to population studies, FM has a moderate to severe impact on individuals in the Spanish population, as measured by the Fibromyalgia Impact Questionnaire (FIQ), leading to significant disability among those affected [6,7]. Research in FM presents a challenge to the scientific community, as no specific pathophysiological mechanism responsible for this disease has been identified. Nevertheless, the relationship between neuroinflammation and the presence of FM is increasingly prevalent in the scientific literature as a promising etiopathogenesis [8].

Various alterations in the transmission, processing, and modulation of pain have been recognized in this population, which are characteristic of chronic pain [9,10,11,12]. These alterations in the neurophysiological mechanisms of pain are believed to lead to an imbalance between pain signal facilitation and inhibition, favoring pain transmission and resulting in a state of central sensitization [13]. Dysfunction of analgesic mechanisms in FM patients is considered a significant biomarker in the pathophysiology of this syndrome. Diffuse noxious inhibitory controls (DNIC) are a mechanism that typically reduces the perception of a painful stimulus when another painful stimulus is applied to a different part of the body. In FM patients, this mechanism is significantly impaired, indicating a failure in descending pain modulation [14]. Another finding that highlights the dysfunction of inhibitory mechanisms is the reduced number of opioid receptors in brain areas responsible for pain modulation [15]. Additionally, inhibitory neurotransmitters like gamma-aminobutyric acid (GABA) play a crucial role in suppressing pain signals. In FM, decreased GABA activity has been observed, which may contribute to the loss of inhibitory control over pain signals, thereby intensifying the experience of chronic pain [16].

Conditioned pain modulation (CPM) is a test that evaluates the functioning of the analgesic system. CPM occurs when the introduction of a second noxious stimulus (conditioning stimulus) reduces the perceived pain from an initial stimulus (test stimulus) applied to a contralateral area [17]. This process suggests that the pain signal processing inhibits nociceptive input from a different region. Therefore, deficits in the CPM mechanism could result in an enhanced perception of pain [18]. CPM is considered a process mediated at the spinal level, involving cortical regions and brainstem structures such as the anterior cingulate cortex and periaqueductal gray [19]. Evidence from animal studies suggests that CPM is partially driven by DNIC [20]. Therefore, descending pain-inhibitory networks play a key role in modulating CPM, and disruptions in these pathways may lead to heightened pain sensitivity. Various chronic pain populations, including FM patients, have shown alterations in CPM [21]. One of the gaps in current knowledge is determining which protocols are most suitable for different chronic pain populations when establishing the CPM paradigm. For instance, the type of conditioning stimulus used in previous studies can vary, including thermal stimuli (heat or cold), electrical stimuli, or ischemic pressure stimuli. Additionally, the intensity of pain reached can range from mild (3/10 on a numerical pain rating scale (NPRS)) to intense (7/10 on NPRS).

In patients with FM, the previous scientific literature has described correlations between poor sleep quality [22], cognitive dysfunctions [23], and body schema disturbances [24] with impaired functioning of the descending pain inhibitory system. However, according to a literature review, deficits in CPM do not always correlate with clinical manifestations of pain, such as disability or pain intensity [25]. This inconsistency may be due to the significant heterogeneity among the protocols used to assess CPM, or it could also be due to the difficulty of diagnosing FM, as it usually lasted years. The search for a “gold standard” protocol for evaluating CPM is crucial to enhancing the understanding of this mechanism in both clinical and research settings for patients with FM [8,26].

We hypothesize that patients with FM will obtain CPM results that indicate the malfunctioning of the pain inhibitory system and that these will also correlate with a worse clinical status. There is a lack of prior knowledge to determine which CPM paradigm will achieve the greatest effect in patients with FM. The main objective of this study was to compare the results of two paradigms to evaluate CPM in women with FM. The secondary objective was to correlate the results of each CPM paradigm with the clinical status of patients with FM to determine the direction that these variables follow in relation to each other.

## 2. Materials and Methods

### 2.1. Participants

A cross-sectional study was designed following STROBE guidelines [27]. The participants were 103 women diagnosed with FM and were assigned to one of two study groups. In Group 1, CPM was evaluated using a thermal conditioning stimulus involving cold water. In Group 2, CPM was assessed using an ischemic conditioning stimulus with a sphygmomanometer. The patients were recruited from FM patient associations in Madrid, “AFYNSIFACRO” and “AFIBROM”, through local support group adverts and presentations. Data collection took place from September 2021 to December 2023, approved by the Rey Juan Carlos University Ethical Review Board (2605202012920 and 1601202303523) in line with the Declaration of Helsinki. Convenience sampling was employed. Convenience sampling and recruitment through local groups were chosen for their practicality and efficiency, allowing quick and cost-effective access to participants. All participants gave written informed consent. Inclusion criteria for FM patients were: (1) FM diagnosis by a rheumatologist, (2) pain for over three months, (3) fluency in spoken and written Spanish, and (4) they were more than 18 years old. A researcher ensured participants understood the tasks and answered questions about the self-administered questionnaires. Exclusion criteria for FM patients included the cognitive inability to understand or complete measurement variables, having or having had cancer, psychiatric disorders, or other major illnesses, such as Raynaud’s syndrome, Lyme, Hepatitis, or irritable bowel. Psychiatric disorders such as schizophrenia or bipolar disorder were excluded, as they could introduce bias in the self-administered questionnaires

### 2.2. Pain Measures and Clinical Status

Pain intensity was measured using the Numeric Pain Rating Scale (NPRS), a validated and widely used tool for self-reported pain assessment in people with chronic pain. The NPRS is an 11-point scale where 0 represents no pain and 10 represents the worst possible pain. NPRS scores are interpreted as follows: 0 = no pain, 1–3 = mild pain, 4–6 = moderate pain, and 7–10 = severe pain. Recent studies strongly recommend the NPRS for its high sensitivity and consistent measurement of pain. [28].

The impact of FM on daily functioning and disability was assessed using the Spanish version of the FIQ. This questionnaire includes 10 items evaluating how FM symptoms affected daily functioning over the past week. The first four items assess physical function, well-being, and work performance using Likert-type scales. The remaining items use a 10 cm visual analogue scale (VAS) to measure pain intensity, fatigue symptoms, sleep quality, stiffness, and the psychological symptoms of anxiety and depression. Likert scores are converted to a 0–10 range, and VAS scores are used directly. The global score is calculated by summing the individual scores, ranging from 0 to 100. Scores of 0–39 indicate mild impact, 40–59 indicate moderate impact, and 60–100 indicate severe impact. This questionnaire has been validated amongst the Spanish population, showing high sensitivity, specificity, and internal consistency [6,29].

Mechanical hyperalgesia was assessed using the pressure pain threshold (PPT). The PPT refers to the minimum pressure required to elicit a sensation of pain under standardized conditions. It is a reliable measure of pain sensitivity. A digital algometer (Model FXD 10, Wagner Instruments, Greenwich, CT, USA) measured pressure in kg/cm^2^ on the right upper trapezius muscle. Pressure was increased at a rate of one kilogram per second until pain was reported. Two measurements were taken with a 30-s interval, and the mean value was recorded as the final result. This method has shown high intra-examiner reliability (ICC = 0.97) and substantial inter-examiner reliability (ICC = 0.79) for the upper trapezius in a healthy population. In FM patients, the method was validated for assessing mechanical hyperalgesia, with an ICC of 0.88. The PTT results below 4 kg/cm² indicate mechanical hyperalgesia [30,31,32]. Also, the PPT was selected as the test stimulus because pressure stimuli are considered more reliable than other stimuli such as heat pain thresholds. Moreover, the CPM effect is best interpreted using pain thresholds and stimuli of predefined intensity as a test stimulus [33,34].

### 2.3. Conditioned Pain Modulation (Cold Stimulus) 

The cold pressor test protocol followed these steps: first, a PPT measurement was taken on the upper region of the right trapezius muscle, as previously described. Next, the participant’s left arm was submerged in a constant-temperature water bath at 12 °C for 60 s, or until a pain intensity of 7/10 was reported on the NPRS. Following the cold exposure, a second PPT measurement was taken on the same location of the right trapezius muscle. The CPM value was calculated by subtracting the PPT value obtained during the conditioning stimulus (the cold water immersion) from the PPT value measured without the conditioning stimulus. In healthy individuals with a well-functioning descending inhibitory pain system, the second algometry measurement is expected to show increased pressure tolerance compared to the initial measurement before reaching the pain threshold. This indicates that the conditioning stimulus enhances pressure tolerance. In populations with chronic pain, however, this pain modulation system may not function as efficiently. The cold pressor test, used as a conditioning stimulus, has demonstrated good intra-session reliability in chronic pain populations (ICC = 0.77), supporting its utility as a valid tool for assessing pain modulation in these groups [35].

### 2.4. Conditioned Pain Modulation (Ischemic Stimulus)

The CPM paradigm with an ischemic conditioning stimulus was established as follows. First, the PPT of the right upper trapezius muscle was measured. To apply the ischemic conditioning stimulus, a sphygmomanometer was used on the left arm and inflated to a constant pressure of 240 mm Hg until a perceived pain intensity of 7/10 on the NPRS was reached. Previous data support the view that using a contralateral dermatome provides the most reliable results [36]. Immediately afterwards, the PPT of the right upper trapezius muscle was re-measured. The CPM result was calculated by subtracting the post-conditioning stimulus value from the initial value. Negative or near-zero values are interpreted as indicating a dysfunction in the pain inhibitory system, which is characteristic of patients with chronic pain. Ischemic pressure as a conditioning stimulus has shown favorable intra-session reliability (ICC = 0.51) [35]. 

### 2.5. Statistical Analysis

Data were analyzed using the Statistical Package for the Social Sciences (SPSS, version 28.00, IBM, Chicago, IL, USA). A 95% confidence interval (CI) was applied, and statistical significance was set at *p*-values below 0.05. Comparisons of nominal variables, such as profession and marital status, were conducted using the Chi-square test across the different groups. Normality was assessed using the Shapiro–Wilk and Kolmogorov–Smirnov tests, which indicated that the sample did not follow a normal distribution set. The PPT after the conditioning stimulus, and CPM variables between groups, were compared using the non-parametric Mann–Whitney U test for independent samples, as the data did not follow a normal distribution. The correlation between conditioned pain modulation variables and clinical measures (PPT, FIQ, and NPRS) was assessed using Spearman’s Rho correlation coefficient. A coefficient greater than 0.60 was interpreted as a strong correlation, values between 0.30 and 0.60 as a moderate correlation, and values below 0.30 as a weak correlation. A significance level of *p* < 0.05 was applied to all statistical tests during the analysis.

## 3. Results

### 3.1. Clinical Status

Group 1 (cold pressor CPM) consisted of fifty women with FM with a mean age of 51.92 ± 7.76 years, while Group 2 (ischemic CPM) comprised fifty-three women with FM, with a mean age of 50.94 ± 8.43 years. According to the FIQ scores, patients in both groups exhibited a severe impact of the disease, with mean scores of 86.36 ± 3.64 in Group 1 and 73.42 ± 13.30 in Group 2. Both groups also reported high pain intensities on the Numerical Pain Rating Scale (NPRS), with a mean of 6.14 ± 1.72 ± 1.51 in Group 1 and 7 in Group 2. Additionally, both groups demonstrated mechanical hyperalgesia as measured by the pressure pain threshold (PPT) in the right upper trapezius muscle, with mean scores of 2.15 ± 0.39 in Group 1 and 3.01 ± 2.07 in Group 2. The results can be seen in Table 1.

### 3.2. Pressure Pain Threshold after Conditioning Stimulus

After receiving the conditioning stimulus (cold pressor in Group 1 and ischemic pressure in Group 2), the subsequent measurement of the pressure pain threshold (PPT) in the right upper trapezius muscle indicated that both groups continued to exhibit mechanical hyperalgesia. The mean scores were 2.39 ± 0.45 for Group 1 and 2.67 ± 2.14 for Group 2. Statistical analysis showed no statistically significant differences between the groups for this variable (*p* = 0.276). The results can be seen in Table 2

### 3.3. Conditioned Pain Modulation

The independent sample Mann–Whitney U-test revealed significant inter-group differences. Both groups obtained values close to zero or negative in the CPM (0.22 ± 0.23 for Group 1 and −0.33 ± 0.71 for Group 2), which is considered indicative of the malfunctioning of the pain inhibitory system. Statistically significant differences were found in the final CPM values between groups (*p* < 0.001). The results can be seen in Table 3.

### 3.4. Correlation Analysis

After examining the bivariate relationships between conditioned pain modulation paradigms and clinical status variables, statistically significant correlations were found. The correlation found in group 1 suggests that women with FM with greater pain intensity in the NPRS and higher disability indices in the FIQ obtain worse results in the CPM with a cold pressor test conditioning stimulus. The results can be seen in Table 4.

## 4. Discussion

The main objective of this study was to compare the results of two paradigms to evaluate CPM in women with FM. The secondary objective was to correlate the results of each CPM paradigm with the clinical status of patients with FM to determine the direction that these variables follow in relation to each other.

### 4.1. Differences in Conditioned Pain Modulation Paradigms in Women with Fibromyalgia

Both study groups demonstrated a similar clinical profile, characterized by a severe impact of the disease, high pain intensities, mechanical hyperalgesia, and a deficit in the functioning of the descending pain inhibitory system. Initially, given the lack of statistically significant differences in the PPT measurement following the conditioning stimulus, we assumed that there would be no differences between the conditioning stimuli used to establish the CPM paradigm. However, further analysis of the final CPM values revealed statistically significant differences between the groups. Group 1, which received the cold pressor conditioning stimulus, exhibited positive values close to zero. In contrast, Group 2, which received the ischemic pressure conditioning stimulus, showed negative or near-zero positive values. Both outcomes indicate a dysfunction in the pain inhibitory system. In a healthy population, the expected result for the final CPM value would be positive, indicating a higher PPT tolerance after the conditioning stimulus. According to our findings, the cold pressor conditioning stimulus elicited a significantly greater response in activating the pain inhibitory system compared to the ischemic pressure stimulus. Additionally, in secondary analysis, the CPM with the cold pressor stimulus showed a correlation with NPRS and disease impact (FIQ). This correlation suggests that patients reporting higher NPRS and FIQ scores tend to achieve lower scores in the CPM paradigm with the cold pressor conditioning stimulus.

Alterations in CPM are characteristic of various chronic pain populations, typically showing reduced activity in the pain inhibitory system following a conditioning stimulus. The scientific literature on CPM is highly heterogeneous, with significant variability in how the paradigm is established. Differences include the type of conditioning stimulus used (thermal, pressure, electrical), the anatomical region assessed for PPT (e.g., upper trapezius muscle, first metacarpal, or anterior tibial muscle), the duration of exposure to the painful conditioning stimulus, and the pain intensity required to activate the descending inhibitory system [37,38,39].

In our study, we chose the cold pressor conditioning stimulus due to its superior intraclass correlation coefficient (ICC) in previous studies involving chronic pain patients. We also included the ischemic pressure stimulus using a sphygmomanometer, as it is a low-cost CPM assessment method, though its efficacy in chronic pain populations is less well established [35]. The right upper trapezius muscle was selected as the PPT reference point because its measurement characteristics have been extensively studied [30]. The pain intensity threshold for the conditioning stimulus was set at 7/10 on the NPRS to ensure a high-intensity level [28]. Like our findings, other studies on FM patients have investigated CPM and obtained comparable results. However, some of these studies have employed different methods to establish the CPM paradigm [14]. For example, in the study by Potvin et al. [40], the cold pressor conditioning stimulus was also used, but the pain threshold measurements were conducted using a thermal heat stimulus. In the study by Coppieters et al. [23], a sphygmomanometer was used to apply the ischemic pressure conditioning stimulus. Unlike our study, patients were required to report a pain intensity of 3/10 on the NPRS while receiving the conditioning stimulus. Additionally, their study did not find an impairment in the descending pain inhibitory system, possibly due to the lower intensity of the conditioning stimulus. 

Regarding the water temperature variable in the cold pressor test, there are also differences in the previous scientific literature. In the study by Caumo et al. [41], water was maintained at a constant temperature of 0° Celsius until a pain intensity of 60/100 was achieved. Additionally, unlike our study, the anatomical region evaluated for the CPM outcome was the right forearm. The research by Chalaye et al. [42] yielded results like ours and used a water temperature of 12° Celsius. The difference was the level of pain intensity required in their study (60/100 on NPRS) and the anatomical region evaluated for the CPM outcome, which was the left forearm. Additionally, our study has a larger sample size, which broadens the current understanding of this aspect of CPM in FM. 

In relation to the correlation between CPM and clinical status, our findings align with the recent study by Gil-Ugidos et al. [43] concerning CPM measurements using the ischemic pressure conditioning stimulus and the correlation with the FIQ and NPRS questionnaires. However, their study did not evaluate CPM using the cold pressor conditioning stimulus. Another difference is that they assessed the PPT in the extensor carpi radialis longus muscle, whereas we evaluated the PPT in the upper trapezius muscle.

The findings of our study, which align with previous scientific literature, may be attributed to the use of the cold pressor test at a water temperature of 12 °C, where the conditioning stimulus reaches a severe pain intensity of 7/10 on the NPRS. Additionally, the PPT was assessed in the upper trapezius muscle, an anatomical region known for its high inter-observer reliability in evaluation. In contrast, previous studies that did not find alterations in CPM may have used a conditioning stimulus that evoked a mild pain sensation of 3/10 on the NPRS and assessed more complex anatomical regions for PPT evaluation. Recent studies suggest that gut dysbiosis may contribute to systemic inflammation, which could affect pain modulation in chronic pain conditions like fibromyalgia. For example, microbial products such as lipopolysaccharide (LPS) have been associated with increased pain and inflammation, indicating a potential link between the gut microbiome and the exacerbation of central sensitization mechanisms in fibromyalgia patients [44].

### 4.2. Implications for Clinical Practice

The findings of this study suggest several important considerations for clinical practice. Both cold pressor and ischemic pressure stimuli are valid methods for assessing the capacity of the pain inhibitory system in women with FM. However, the cold pressor conditioning stimulus appears to be more effective at establishing the CPM paradigm, as it triggers a stronger response and more effectively activates descending pain pathways. This is crucial for evaluating the efficacy of pain modulation strategies in clinical settings.

Despite this, clinicians should be aware that the cold pressor test may not be feasible for FM patients with certain comorbid conditions, such as Raynaud’s syndrome. In such cases, the ischemic pressure stimulus, which only requires a sphygmomanometer, offers a more accessible and less costly alternative, even though it may be less effective. Thus, the use of the ischemic pressure stimulus should not be dismissed, particularly for monitoring treatment effects and pain modulation in FM patients [45].

The regular use of CPM paradigms can also serve as a potential predictor for the progression of pain in FM patients. Incorporating CPM testing into clinical practice allows for a more tailored approach to pain management, enabling proactive adjustments to treatment plans based on real-time assessments of the pain inhibitory system’s functionality. This integration bridges the gap between research and practice, providing robust data to support clinical decision-making. It is recommended that healthcare professionals be trained in the application and interpretation of CPM results to enhance overall FM management and optimize patient outcomes.

However, the limitations identified in this study highlight the need for the careful interpretation of these findings. The potential lack of clinical relevance of the statistically significant differences observed suggests that clinicians should exercise caution when using FIQ scores alone to distinguish between levels of disease severity in FM patients. The influence of ongoing medication use and potential measurement bias further indicates the necessity for additional research under more controlled conditions. Including a healthy control group in future studies could improve the generalizability of results and better define the clinical utility of various pain modulation assessments in FM management.

### 4.3. Future Research Lines

The following research directions are proposed: design randomized clinical trials to evaluate changes in CPM after applying treatment techniques; conduct longitudinal studies with repeated CPM measurements over time to assess how these changes influence the clinical state of women with FM; and perform cross-sectional studies to compare CPM paradigms between FM and other chronic pain populations, such as chronic lower back pain, complex regional pain syndrome, or whiplash-associated disorders, to identify potential differences in this pathophysiological mechanism within central sensitization syndromes [46,47]. Future studies should also focus on a deeper analysis of FM patients who are responders and non-responders to CPM paradigms. Subclassifying these subjects into two groups could help to understand the relationship between CPM responses and other clinical variables.

### 4.4. Limitations

This study has several limitations. Firstly, although there were statistically significant differences in the FIQ scores between the two groups (*p* < 0.001), both groups demonstrated severe disability based on these scores. This finding suggests that, despite statistical significance, the observed differences may lack clinical relevance, as both groups are categorized within the same severity level. This potential lack of clinical significance could be attributed to the high variability in pain perception and functional impairment commonly observed in the FM population. Consequently, these results should be interpreted with caution, acknowledging that statistical significance does not necessarily equate to clinical importance in the context of chronic and heterogeneous conditions such as FM. 

Moreover, the researchers performing the measurements were not blinded to group allocation, which may have introduced measurement bias. Another limitation is the method of participant recruitment; when interpreting the results and extrapolating to the general population, the potential for selection bias must be considered. Additionally, participants continued their regular medication regimens during the study, which could have influenced the outcomes. Lastly, the absence of a healthy control group limits the generalizability of the findings to the broader population.

## 5. Conclusions

Pain modulation abnormalities in FM patients are evident when using either the cold pressor or ischemic pressure stimuli to establish the CPM paradigm. The cold pressor conditioning stimulus elicits a stronger response than the ischemic pressure stimulus in FM patients. Greater impairment of the inhibitory system, as evaluated by the CPM paradigm using the cold pressor stimulus, correlates with higher pain intensity (NPRS) and a higher disability index (FIQ).

## Figures and Tables

**Table 1:** Descriptive statistics in demographic measures (*n* = 103).

Measures	Group 1 (*n* = 50)	Group 2 (*n* = 53)	*p* Value Independent Samples Mann–Whitney U-Test
Age	51.92 ± 7.76	50.94 ± 8.43	0.620
Pain (NPRS)	6.14 ± 1.72	7.00 ± 1.51	0.009
FIQ	86.36 ± 3.64	73.42 ± 13.30	0.001
PPT Pre-stimulus	2.15 ± 0.39	3.01 ± 2.07	0.790

Data are presented as mean (SD). Both FM groups present high intensities of clinical pain intensity on the numeric pain rating scale (NPRS), high levels of impact of the disease measured with the Fibromyalgia Impact Questionnaire (FIQ), and also mechanical hyperalgesia before receiving the conditioning stimulus measured with pressure pain threshold (PPT).

**Table 2:** Comparison between groups in pressure pain threshold after receiving conditioning stimulus measures.

Measures	Group 1 (*n* = 50)	Group 2 (*n* = 53)	*p*-Value Independent Samples Mann–Whitney U-Test
PPT post	2.39 ± 0.45	2.67 ± 2.14	0.276

This table showed differences between groups in psychophysiological measures of PPT pressure pain threshold) after receiving a conditioning stimulus. Both groups obtained results that showed mechanical hyperalgesia. There are no statistically significant differences between groups.

**Table 3:** Comparison between groups in conditioned pain modulation measures.

Measures	Group 1 (*n* = 50)	Group 2 (*n* = 53)	*p*-Value Independent Samples Mann–Whitney U-Test
CPM	0.22 ± 0.23	−0.33 ± 0.71	0.001

This table show differences between groups in psychophysiological measures of CPM (conditioned pain modulation). Both groups obtained results that show the deficit in the functioning of the inhibitory system. There are significant differences in the final values obtained in the CPM.

**Table 4:** Correlation coefficients between conditioned pain modulation paradigms and clinical status variables.

Measures	CPM (Cold Pressor)	CPM (Ischemic Pressure)
NPRS	−0.357 *	−0.075
FIQ	−0.377 *	0.147
PPT Pre	0.15	−0.161

A moderate correlation was found between the CPM cold pressor paradigm, pain intensity (NPRS), and disease impact (FIQ). * Indicates correlation (statistically significant).

## Data Availability

Data are contained within the article.
